# Understanding the barriers to using oral anticoagulants among long-term aspirin users with atrial fibrillation – a qualitative study

**DOI:** 10.1186/s12913-020-05947-3

**Published:** 2020-11-25

**Authors:** Vanessa W. S. Ng, Chung-Wah Siu, Patrick K. C. Chiu, Carolyn P. L. Kng, Elizabeth Jamieson, Ian C. K. Wong, May P. S. Lam

**Affiliations:** 1 Centre for Safe Medication Practice and Research, Department of Pharmacology and Pharmacy, The University of Hong Kong, Hong Kong SAR, China; 2 Cardiology Division, Department of Medicine, Li Ka Shing Faculty of Medicine, The University of Hong Kong, Hong Kong SAR, China; 3 Division of Geriatrics, Department of Medicine, Queen Mary Hospital, Hong Kong SAR, China; 4 Division of Geriatrics, Ruttonjee and Tang Shiu Kin Hospitals, Hong Kong SAR, China; 5 Research Department of Practice and Policy, University College London School of Pharmacy, London, UK

**Keywords:** Barriers, Oral anticoagulants, Aspirin, Atrial fibrillation

## Abstract

**Background:**

Despite international treatment guidelines currently advocating oral anticoagulants (OACs) as the only appropriate stroke prevention therapy for patients with atrial fibrillation (AF) and evidence that OACs can greatly reduce the risk of stroke with similar risk of bleeding compared with aspirin, the underuse of OACs in patients with AF is common globally, especially in Asia. This study aimed to identify the barriers to prescribing and using OACs among long-term aspirin users with AF.

**Method:**

Face-to-face interviews were conducted with fourteen eligible patients with AF using a semi-structured interview guide. The interview recordings were transcribed verbatim and data was analyzed according to the principles of thematic analysis.

**Results:**

Five themes were developed: awareness of AF symptoms and diagnosis; knowledge and understanding of AF and stroke prevention therapy; role of decision-making in prescribing; willingness to switch from aspirin to OACs; and impact of OAC regimen on daily living. The majority of the patients were not aware of the symptoms and diagnosis of AF and only had a vague understanding of the illness and stroke prevention therapy, leading to their minimal involvement in decisions relating to their treatment. Some patients and their caregivers were particularly concerned about the bleeding complications from OACs and perceived aspirin to be a suitable alternative as they find the adverse effects from aspirin manageable and so preferred to remain on aspirin if switching to OACs was not compulsory. Lastly, the lifestyle modifications required when using warfarin, e.g. alternative dosing regimen, diet restriction, were seen as barriers to some patients and caregivers.

**Conclusion:**

The findings revealed patients’ knowledge gap in AF management which may be targeted using educational interventions to improve patients’ understanding of AF and its management and hence encourage active participation in the decision-making of their treatment in the future.

## Background

Atrial fibrillation (AF) is a common cardiac arrhythmia, which is associated with a 5-fold greater risk of ischemic stroke [1]. Stroke caused by AF is associated with a higher risk of hospitalization and disability, creating economic burden to the family and society [2–4]. Therefore, oral anticoagulants (OACs) are vital treatments for stroke prevention in AF patients. OACs, including warfarin and other non-vitamin K antagonist anticoagulants (NOACs), have proven to be effective in greatly reducing the risk of stroke and mortality [5–9]. Since 2018, international treatment guidelines of atrial fibrillation have been updated to reflect the fact that OACs are advocated as the only stroke prevention therapy in AF patients with a CHA_2_DS_2_VASc (congestive heart failure, hypertension, age ≥ 75 [doubled], diabetes mellitus, past history of stroke or transient ischemic attack [doubled], vascular disease, age between 65 and 74, sex [female]) score of ≥2 in men and ≥ 3 in women unless contraindicated [10–14]. Aspirin monotherapy for preventing stroke was no longer recommended for AF patients regardless of their risk of stroke [10–14].

A randomized controlled trial showed warfarin was associated with a lower risk of fatal or disabling stroke among elderly patients with AF compared to aspirin (1.8% vs 3.8%) [15]. Similar findings were observed in a recent study in Hong Kong. OACs were associated with lower risk of ischemic stroke and all-cause mortality compared to antiplatelets but both OACs and antiplatelets demonstrated a comparable risk of gastrointestinal bleeding [16]. These findings support the fact that antiplatelet drugs should not be recommended as first-line therapy for stroke prevention in high-risk AF patients [8, 15–17]. However, the issue of underuse of OACs has been addressed across the globe over the years. A systematic review summarized that the majority of the studies conducted between 1997 and 2008 in various countries reported less than 60% of patients with AF at high risk of stroke were untreated with OACs [18]. With the introduction of NOACs since 2010, it was expected the underuse of OACs would improve. A study in Denmark reported that OACs initiation rate in newly diagnosed AF patients decreased from 46.3% in 2005 to 38.1% in 2009, then increased rapidly to 66.5% in 2015 [19]. In the UK, the use of OACs in patients with AF increased from 54.7% in 2011 to 73.9% in 2016 while the use of antiplatelet drugs declined from 36.4 to 10.5% over the same period [20]. In Hong Kong, the proportion of AF patients receiving antiplatelet therapy was almost twice as those receiving OACs (43% vs 26%) in 2006 [16]. Similarly, the overall rate of antithrombotic therapy was 37.7%, with the use of aspirin and warfarin being 32.3% and 4.1% respectively in China [21]. The rate of aspirin use increased around 8–10 times from 2007 to 2012 while the uptake of warfarin only increased less than twice in 4 years, reflecting the underuse of OACs was more common in Asian countries and Chinese patients with AF were more likely to be prescribed aspirin as aspirin is still often perceived as a safer alternative to OACs [21, 22]. Therefore, understanding the barriers to prescribing and the underlying reasons for underuse of OACs for stroke prevention in AF patients is important to ensure their safety and quality of care.

Previous studies reported some potential factors explaining the underuse of warfarin in patients with AF, namely understanding of AF and stroke prevention management, risk and benefits of warfarin therapy, treatment decision-making and any challenges of treatment management when compared to NOACs [23, 24]. However, these studies were conducted in western countries. Ethnicity might affect the perception of patients using OACs. As shown from previously published studies, Asians have higher risks of intracranial hemorrhage compared to Caucasians [25, 26]. The current findings might not fully explain the phenomenon of under-prescribing OACs in Hong Kong.

To the best of our knowledge, no study has explored the perception of patients with AF regarding the use of OACs versus aspirin for stroke prevention. Our study aimed to identify the barriers to prescribing and using OACs among long-term aspirin users with AF.

## Method

### Recruitment

Participants were eligible to participate in this study if they were diagnosed with AF, had been taking low dose aspirin including combination with clopidogrel for more than 12 months and were not current users of OACs. Patients were excluded if they were diagnosed with other heart diseases that require low dose aspirin as part of the routine treatment, such as acute coronary syndrome, cardiac hypertrophy, myocardial infarction, coronary artery disease, were prescribed aspirin before a diagnosis of AF and were unable to communicate in Cantonese or English. Purposive sampling was used to recruit participants. Weekly out-patient clinic lists were obtained from the Division of Geriatrics from Queen Mary Hospital (QMH) and Ruttonjee and Tang Shiu Kin Hospital (RTSKH). Potential participants who met the selection criteria were identified by a researcher (VN) screening from the clinic lists via Electronic Patient Records authorized by the Hong Kong Hospital Authority. Eligible patients and their caregivers who accompanied the patients to out-patient appointments were invited to attend face-to-face interviews before or after the doctors’ consultations at the geriatric and memory clinics at the respective hospital sites. Patient information leaflets and a written consent form prepared by two researchers (VN and ML) were given for their considerations. Ethics approvals were sought and obtained from the Institutional Review Board of the University of Hong Kong/Hospital Authority Hong Kong West Cluster (UW18–580) and Hong Kong East Cluster Research Ethics Committee (HKECREC-2019-007).

### Procedure

Face-to-face interviews were carried out in consultation rooms at QMH Main Pharmacy and RTSKH. The semi-structured interview guide was developed by two researchers (VN and ML) and validated by cardiologists (CWS), geriatricians (PC and CK), and pharmacists (IW and ML) to ensure the content of the guide was clinically relevant [see Additional file 1]. The interview guide included questions designed to explore patients’ experience of diagnosis of AF, understanding of illness and stroke prevention treatment, and any factors that might affect the decision of doctors prescribing stroke prevention therapy. Written informed consent was obtained by the researcher (VN) prior to the interview. The interviews were conducted with patients and/or caregivers and audiotaped with patients’ permission by the same researcher (VN). Field notes were also made on interviewees’ emotions, facial expressions and speaking tones during the interviews. After the interview, an HKD 100 supermarket voucher was awarded to each participant as a token of thanks.

### Data analysis

All interview recordings were transcribed verbatim and anonymized. Data was then analyzed according to the principles of thematic analysis, which is an inductive and comparative process of collecting, categorizing by codes, analyzing and conceptualizing the series of codes to identify new patterns from qualitative data [27]. Each transcript was coded line by line and codes with similar meanings or implications were grouped together to form sub-themes. Data collection and analysis were conducted concurrently so any themes identified from existing data could be followed up when interviewing new patients [28]. Several meta-themes were developed after systematic and repetitive analysis on the sub-themes. Data collection stopped when data saturation was achieved, i.e. where no more new themes were identified. NVivo (QSR International Pty Ltd., Version 12, 2019, Victoria, Australia) was used to facilitate the identification and refinement of patterns and themes. Data was analyzed by two researchers (VN and ML) independently and mutual agreement was reached on coding and themes.

## Results

### Patients characteristics

**Fig. 1:**
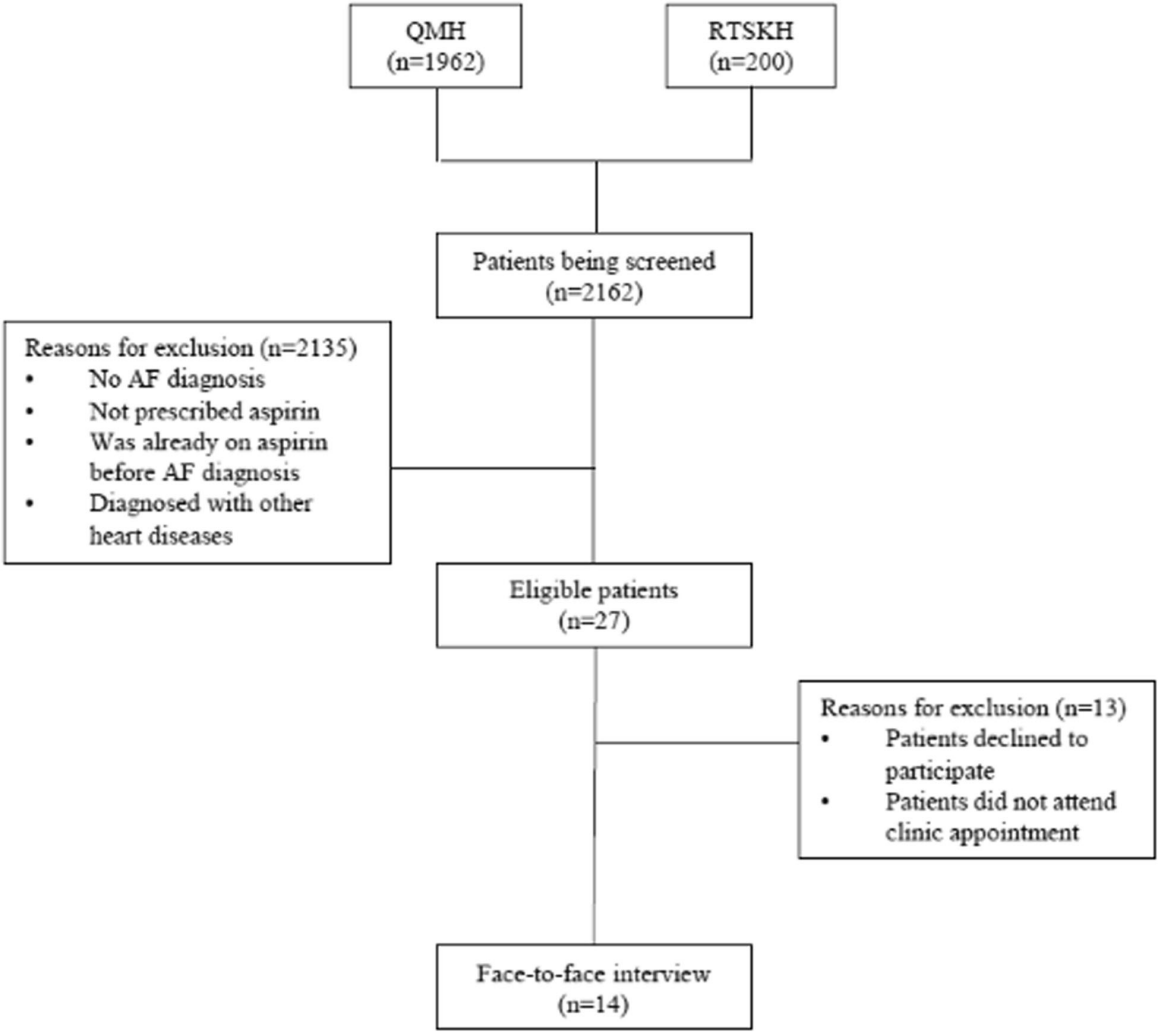
Flowchart of patient selection

Of the 2162 individuals scheduled to attend their out-patient appointments between 1st January 2019 and 31st August 2019 at the clinics in both QMH and RTSKH (1962 from QMH and 200 from RTSKH respectively), 27 fulfilled the inclusion criteria and 14 of them agreed to be interviewed (Fig. 1). The caregivers of 10 out of 14 patients agreed to be interviewed and they were interviewed as a pair.

All patients had a CHA_2_DS_2_-VASc score of ≥2 and 85.7% of them had a HAS-BLED score between 0 and 2. The characteristics of patients are summarized in Table 1. Data saturation was reached after interviewing 14 patients. The interviews lasted approximately 15 min.

**Table 1:** Patient characteristics

Patients Characteristics	Participants, n (%)
**Gender**	
Males	4 (28.6)
Females	10 (71.4)
**Mean age at the first AF diagnosis (SD)**	81.9 (8.6)
**Mean age at the time of interview (SD)**	87.7 (4.8)
**Level of education**	
No schooling	5 (35.7)
Primary	5 (35.7)
Secondary	2 (14.3)
Post-secondary	2 (14.3)
**CHA**_**2**_**DS**_**2**_**-VASc Score**	
0–1	0 (0)
2–3	5 (35.7)
4–6	7 (50.0)
7–9	2 (14.3)
**HAS-BLED Score**	
0–2	12 (85.7)
3–5	2 (14.3)
6–9	0 (0)

Abbreviations: *AF* Atrial fibrillation, *SD* Standard deviation

Five meta-themes were developed: Awareness of AF symptoms and diagnosis; Knowledge and understanding of AF and stroke prevention therapy; Role of decision-making in prescribing; Willingness to switch from aspirin to OACs; and Impact of OACs on daily lives.

#### Theme 1: awareness of AF symptoms and diagnosis

More than half of the patients and their caregivers were not aware of symptoms of AF until they were incidentally diagnosed during routine examinations or when complications of AF happened. (“*I couldn’t tell if my heart actually beats irregularly but doctor found out after ECG.”* ***P10****; “We realized that Grandma got AF after stroke, and she did not take any medications before.”* ***Caregiver of P06****).* The remaining patients did not recall the fact they had been medically diagnosed with AF.

#### Theme 2: knowledge and understanding of AF and stroke prevention therapy

i. **Understanding of AF and complications**

The majority of the patients had a poor understanding of AF and its complications. They were able to describe AF as having an irregular heartbeat and palpitations without knowing the name of the disease. (*“I don’t know the name (AF). The doctor only told me my heartbeat is not good every time.”* ***P12***; *“What I know from the doctor is heart rhythm is not regular and fast heartbeat.”* ***Caregiver of P07***). Some patients and their caregivers also mentioned they could not recall what they had been told by the doctors as doctors might not discuss patients’ heart conditions at every consultation. Only three patients knew that AF increases the risk of having a stroke.

ii. **Understanding the importance of stroke prevention**

Understanding the importance of stroke prevention therapy is an important facilitator of using OACs. Nine of our fourteen patients and their caregivers could clearly explain that aspirin and other OACs reduce the formation of blood clots which cause blockage of blood vessels but only two of them could state the linkage to stroke prophylaxis. However, some patients did not know why aspirin was prescribed and some even had an incorrect understanding of the reasons for taking aspirin regarding to AF. *(“Aspirin is used to make the blood become thicker and I’ll be less likely to bleed.”* ***P13****; “Aspirin is to slow down the heart rate.”* ***Caregiver of P07****).* Furthermore, most patients and caregivers described aspirin as a “*blood thinning agent*” but they had no knowledge of other OACs available on the market. (*“Yes, I only know aspirin. I’m not sure about others (anticoagulants) so I didn’t ask the doctor.”* ***P05***). Only the caregiver of one patient was able to illustrate a good understanding of the illness, the importance of stroke prevention therapy, and the difference between OACs and aspirin.

#### Theme 3: role of decision-making in prescribing

i. **Involvement in discussions with doctors**

Patients and caregivers assumed a passive role when communicating with doctors. Generally, there was minimal or no patient involvement in treatment decisions. Most of the time the decisions regarding treatment were made by the doctors and patients accepted their decisions. (*“The doctor only told us Grandma has AF and also hypertension so he prescribed aspirin.”* ***Caregiver of P08***; *“Doctor didn’t say anything but just prescribed aspirin.”* ***Caregiver of P02***). A few mentioned that insufficient medical knowledge is also a barrier to determine which treatment option is more suitable for patients. As mentioned above, most patients had no knowledge of other OACs apart from aspirin. This restricted them from contributing to a treatment decision with doctors. (*“Doctor can decide and I don’t know much on this (medication), as long as it’s good to Grandma.”* ***Caregiver of P09***). The caregiver of one patient mentioned that it was difficult to have an in-depth discussion with the doctors due to limited consultation time. (*“Consultation time is very short at the hospital. The doctor will only call you unless there’s something urgent.”* ***Caregiver of P02***).

ii. **Delegating trust to their doctors**

Patients trusted their doctors to decide what medications should be prescribed for their condition because of their professional knowledge and judgement. (*“I don’t know what doctor prescribed. I trust the doctor!”* ***Caregiver of P04****; “I trust the doctor… I don’t know the medication and I assume the doctor is confident to prescribe the right medication to my Mum. Is that right? Then I trust the doctor.”* ***Caregiver of P11***)*.*

#### Theme 4: willingness to switch from aspirin to OACs

i. **Fear of adverse effects from OACs**

Patients and caregivers expressed their concerns of the adverse effects of OACs compared to aspirin, predominantly the risk of bleeding. They were particularly concerned about potential bleeding complications and fearful about what might happen, leading to them to reject OACs. (*“I dare not take it (OAC). I don’t want to take it. It will cause non-stop bleeding! I’m scared!”* ***P10***). Due to the fragility of elderly patients, their caregivers were worried that the potential of bleeding easily from OACs would increase risk of injuries and hence adversely impact patients’ quality of life. (*“We need to consider if we let Mum take it (OAC) or not. My mum always scratches herself and bleeds easily. The doctor told me about the problem of bleeding. I’m afraid the wounds will get inflamed easily…… Also, if she falls at home, it takes a long time for the bruises to disappear.”* ***Caregiver of P02***; *“I’m worried that if the ‘blood thinning’ of OAC is stronger than aspirin, Mum will faint easily. I don’t agree taking OAC.”* ***Caregiver of P03***)

ii. **Risk assessment of aspirin and OACs**

Most patients and their caregivers perceived that aspirin is a suitable medication for them if they do not experience any adverse effects or find them manageable. (*“I’m fine with taking aspirin so I won’t consider changing to another new one.”* ***P10***) For older patients, their caregivers expressed concerns that it would be difficult for older people to adapt to new medications including dosing regimen, lifestyle modifications and most importantly, the tolerability of unknown adverse effects. Therefore, most patients and caregivers felt the risks of experiencing any unknown adverse effects that they might be unable to tolerate the outweighed risk of having a stroke. (*“Since Mum is old, her body function starts to deteriorate slowly. If the doctor always changes her medication, I’m afraid she won’t be able to adapt”* ***Caregiver of P04***). Only the caregiver of one patient placed more value on the benefits of stroke prevention of OACs and would consider switching from aspirin to OAC in the future.

#### Theme 5: impact of OAC regimen on daily living

Patients and caregivers believed that the initiation of OACs would be inconvenient in their day-to-day lives. Compared to aspirin, the regimens of OACs are more complicated, in which regular drug monitoring, diet restrictions and occasional adjustment of dosage would be required. (*“The doctor was planning to prescribe warfarin but……. Grandma will need to do blood tests every few days and then adjust the dose again. She is pretty old now so aspirin is much simpler. It’s better for her……… Also, she doesn’t need to avoid eating some of the food for aspirin but warfarin.”* ***Caregiver of P04***) One patient experienced gastrointestinal bleeding after taking NOAC, hence aspirin was prescribed after the cessation of NOAC.

## Discussion

Patients with AF can exhibit signs and symptoms ranging from vague, subtle symptoms, such as fatigue or lightheadedness, to severe symptoms such as palpitations or chest pain. Chan and Choy reported 65.3% of patients with newly diagnosed AF were asymptomatic [29]. Most often, these asymptomatic patients were incidentally diagnosed during routine examinations or when AF complications happened, such as stroke [14]. Moreover, symptomatic patients misinterpreted or failed to recognize their AF symptoms and attributed the symptoms to other health conditions (such as hypertension, respiratory disease) or non**-**illness related reasons (such as, stress or ageing) [30]. Wilson et al. reported that 69% of patients described the symptoms were vague and outside of their awareness [31]. This was also found in our study, as the majority of our patients failed to recognize the symptoms and were diagnosed with AF after stroke or during routine checkups.

Lack of knowledge regarding AF, the signs and symptoms and the associated risks have been well documented. A study in the UK reported that only half of the patients were able to name their heart condition and only 57% of patients were aware of the reason for taking an OAC [32]. Similarly, Lip et al. found that only 63% were aware of their cardiac condition and only 52% understood the rationale behind initiating OAC therapy [33]. From our study, almost all the patients were unaware of AF and its signs and symptoms. Compared to the results from previous studies [32, 33], patients in Hong Kong had a weaker understanding about AF. In our study, more than 70% of them had low educational level which is associated with lower health literacy, hence leading to poor understanding about their illness [34]. The results of our study echoed the results from Lee et al. in which they reported none of the patients with AF were aware of their cardiac condition and half of them did not recognize the symptoms of AF [35].

In terms of the rationale of using stroke prevention medications, more than half of our patients understood that aspirin and the OACs were used to prevent “blood clots”, with majority of them unable to state the link between use of aspirin and OACs and stroke prophylaxis. This knowledge gap was also reported in several studies. Whilst the majority of patients could state the reason for taking OACs, many failed to recognize the potential risk reduction of stroke from taking OACs [32, 36]. Insufficient knowledge regarding AF means patients do not appreciate the link between the illness and necessity for stroke prevention therapy. Many patients and their caregivers misinterpreted that aspirin is indicated for AF and hence underestimated the importance of stroke prevention, supporting previous qualitative evidence [23].

Once patients were diagnosed with AF, they would decide whether to initiate stroke prevention therapy and which medication to be most suitable with the doctors. Shared decision making only happened when patients had adequate knowledge about their diseases. Lack of understanding their own disease and treatment options potentially leads to minimal involvement in decision-making and hence total reliance on physicians’ decisions. Therefore, it is not surprising that all our patients were passively involved in their treatment decision making. Many studies have reached similar conclusions. Thrysoee et al. reported patients in their study did not understand the association between AF, stroke, and anticoagulation so they were not actively involved in the decision-making process and took the medication as prescribed by the physician [37]. In addition, a systematic review previously reported that patients’ high level of confidence in physicians’ professional knowledge and training was one of the reasons for decision delegation [24]. A phrase “doctor knows best” was a theme identified from the qualitative study by Clarkesmith et al. and this embedded the trust patients placed in physicians to make the decision on their behalf [23].

Given the lack of understanding of AF and its management, together with the trust placed in physicians, it was not surprising that our patients were reluctant to switch from aspirin to OACs. Fear of bleeding from OACs was repeatedly given as a reason for not switching to OACs by patients and their caregivers. Due to the actual or perceived risk of bleeding, half of the patients refused switching to an OAC and opted for continuous use of aspirin. Information on the bleeding risk associated with OACs greatly influences the willingness of patients to switch. A previous study demonstrated that patients who perceived themselves at low risk of stroke refused to be on warfarin as they were more concerned about the bleeding risks associated with warfarin [38]. Lahaye et al. also demonstrated that patients with AF were not willing to consider OACs due to the fear of bleeding in spite of the importance of stroke prevention [39]. Another study reported that warfarin users considered regular international normalized ratio (INR) monitoring to be a burden and time-wasting [40]. These concerns from previous studies were raised by our patients. Moreover, some patients were fearful about using OACs that did not require monitoring and with limited availability of antidotes, which did not reflect in our study [40]. Last but not the least, experiences of family and peers can greatly influence patients’ willingness to use OACs [24]. In our study, one caregiver indicated that her husband was on a NOAC and she would choose NOAC over aspirin because of better efficacy for the patient. However, due to the older age and declining renal function of the patient, she remained on aspirin.

We believe that our study findings have significant implications for clinical practice and policy. Our findings regarding the knowledge deficit of patients being the primary reason for minimal involvement of patients in decision-making regarding the treatment options for stroke prevention were consistent with previous studies [23, 24, 37]. Educational interventions by health professionals are highly recommended to the public, especially aimed at patients with AF and their caregivers. The components of educational interventions could include a brief overview of AF and its complications, various treatment options of stroke prevention, risks and benefits of aspirin and OACs. This would enhance patients and their caregivers’ understanding and hence potentially improve their adherence to medications and lifestyle recommendations suggested by their doctors. A multinational randomized trial reported the proportion of OACs use increased by 12% 1 year after an educational intervention was implemented [41]. There was a significant reduction of stroke in the intervention group compared to the control group [41]. Another study also found that higher health literacy is associated with a greater time within the therapeutic range in warfarin users [42], reflecting better management of warfarin therapy and thus facilitates stroke prevention.

## Limitations

There are some limitations in this study. Firstly, the sample size was limited. The recruitment of patients from the geriatric and memory clinics might have an impact on limiting the generalizability of the findings as a significant proportion of patients with severe cognitive impairment might be absent from this study and this might not reflect the general AF population. However, the mean age of first diagnosis of AF is 81.9 years in our study, which is similar to 80.0 years in the AF cohort in previous study using the electronic healthcare database covering patients who have utilized services in public hospitals in Hong Kong [16]. Our study also complements findings from similar studies in this area so our findings could reflect the actual barriers that most AF patients experienced when determining whether aspirin or OACs were prescribed. In our study, all patients had a CHA_2_DS_2_-VASc score of ≥2, indicating that they were all eligible for anticoagulation according to current treatment guidelines [10–14]. Therefore, our findings reflected the barriers of using OACs unrelated to their predicted risk of stroke. Secondly, many patients and caregivers failed to recall the details of discussion with the doctor regarding treatment options as it had taken place some time ago. Thirdly, a few patients had mild cognitive impairment and were unable to answer some of the interview questions so the caregivers answered for the patients or further elaborated on their responses.

## Conclusion

AF is a very common cardiovascular disorder yet poorly understood by patients. Patients were not aware of the signs and symptoms of AF, nor its management. Therefore, patients with AF were rarely actively involved in the decision-making process and did not question physicians’ recommendations. Importantly, educational strategies that target the knowledge gap, and misconceptions surrounding AF and OACs, should be developed and active participation in the decision-making process should be encouraged.

## Supplementary Information

**Additional file 1.** Semi-structured interview guide.

## Data Availability

The data that support the findings of this study are available from Queen Mary Hospital and Ruttonjee and Tang Shiu Kin Hospital but restrictions apply to the availability of these data, which were used under license for the current study, and so are not publicly available. Data are however available from the authors upon reasonable request and with permission of Queen Mary Hospital and Ruttonjee and Tang Shiu Kin Hospital in Hong Kong.

## Abbreviations

AF: Atrial fibrillation
INR: International normalized ratio
NOACs: Non-vitamin K antagonist anticoagulants
OACs: Oral anticoagulants
QMH: Queen Mary Hospital
RTSKH: Ruttonjee and Tang Shiu Kin Hospital
