# Supplementary material for: Understanding the barriers to using oral anticoagulants among long-term aspirin users with atrial fibrillation – a qualitative study
Source: BMC Health Serv Res. 2020 Nov 25;20:1084. doi: 10.1186/s12913-020-05947-3 (PMC7691100; doi:10.1186/s12913-020-05947-3)
Supplement: Supplementary file 1 — Additional file 1. Semi-structured interview guide. [file 12913_2020_5947_MOESM1_ESM.docx]

**Additional File 1: Semi-structure interview guide**

**Basic demographics**

1. What is your current employment status?
2. What is your occupation?
3. What is your educational level?

**Knowledge about atrial fibrillation**

1. What have happened to your heart and made you see the doctor?
2. Do you remember what the doctor said at your first diagnosis? What did the doctor say?
3. What are the complications associated with atrial fibrillation?

**Perception of treatment**

1. Do you know the doctor has prescribed aspirin for you?
2. What benefits can you obtain from taking aspirin?
3. Do you know what kind of medications you are taking for your atrial fibrillation? What are they?
4. Have you experienced any side effects from aspirin? If so, what are they?

**Involvement in decision-making process**

1. Did you discuss with your doctor when he/she decided to prescribe aspirin for you? What factors influenced your decision?
2. Have you heard of other alternatives for stroke prevention? Where did you know about it?
3. What is the difference between the other alternatives and aspirin?
4. Why do you choose aspirin?
5. Have you considered having another conversation with your doctor at your next appointment about stroke prevention after this interview? Why or why not?
